# People Efficiently Explore the Solution Space of the Computationally Intractable Traveling Salesman Problem to Find Near-Optimal Tours

**DOI:** 10.1371/journal.pone.0011685

**Published:** 2010-07-29

**Authors:** Daniel E. Acuña, Víctor Parada

**Affiliations:** 1 Department of Computer Science and Engineering and Center for Cognitive Sciences, University of Minnesota, Minneapolis, Minnesota, United States of America; 2 Departamento de Ingeniería Informática, Universidad de Santiago, Santiago, Chile; Massachusetts Institute of Technology, United States of America

## Abstract

Humans need to solve computationally intractable problems such as visual search, categorization, and simultaneous learning and acting, yet an increasing body of evidence suggests that their solutions to instantiations of these problems are near optimal. Computational complexity advances an explanation to this apparent paradox: (1) only a small portion of instances of such problems are actually hard, and (2) successful heuristics exploit structural properties of the typical instance to selectively improve parts that are likely to be sub-optimal. We hypothesize that these two ideas largely account for the good performance of humans on computationally hard problems. We tested part of this hypothesis by studying the solutions of 28 participants to 28 instances of the Euclidean Traveling Salesman Problem (TSP). Participants were provided feedback on the cost of their solutions and were allowed unlimited solution attempts (trials). We found a significant improvement between the first and last trials and that solutions are significantly different from random tours that follow the convex hull and do not have self-crossings. More importantly, we found that participants modified their current better solutions in such a way that edges belonging to the optimal solution (“good” edges) were significantly more likely to stay than other edges (“bad” edges), a hallmark of structural exploitation. We found, however, that more trials harmed the participants' ability to tell good from bad edges, suggesting that after too many trials the participants “ran out of ideas.” In sum, we provide the first demonstration of significant performance improvement on the TSP under repetition and feedback and evidence that human problem-solving may exploit the structure of hard problems paralleling behavior of state-of-the-art heuristics.

## Introduction

We usually take for granted our capacities for vision, motor control, and decision-making under uncertainty, without realizing how computationally demanding these tasks may be [1]–[4]. Any cursory examination of the resources needed to solve these tasks would most likely reveal NP-Complete computational complexity [5]. This term denotes a class of so-called “intractable” problems whose solutions can be checked for correctness in polynomially-bounded time, but finding the optimal solution would require an exponential amount of time in the worst case (the hardest instance) [5]. There is growing evidence, however, that humans find optimal or near optimal solutions to instantiations of these hard problems [6]–[8]. Although finding near optimal solutions may not necessarily involve solving NP-complete problems, the consistency with which humans conform to computationally optimal principles is intriguing. The strong connection between the computational and physical worlds (e.g., see [9]) renders this apparent paradox relevant to understanding how humans—and potentially other animals—are so well prepared to deal with computational intractability.

A similar disconnection between the theoretical intractability of problems and the practical performance of state-of-the-art heuristics has led complexity theorists to develop more refined analyses of hardness than those of worst-case complexity. These refined analyses show that really hard instances seem to be rare in practice and, hence, heuristic optimization specializes on solving well the “typical” (i.e., non-artificial) instances [9]–[13]. There have been two main ways to incorporate this instance-tune analysis into complexity theory. One approach formally defines a richer family of complexity classes, but sacrifices the straightforward application of worst-case intractability—e.g., average-case complexity [14] requires a representative distribution over instances that may be hard to specify, smoothed analysis [13] is difficult to apply to discrete problems, and parameterized complexity [15] requires a non-trivial new dimension (parameter) of problem complexity.

Another approach, more appropriate for the purpose of our paper, is to start from successful heuristics as a key to understand the elements of good performance and to characterize instance hardness. A key result in this approach has been the discovery of hidden structures within instances that, once revealed, exponentially simplify the solution time [12]. Not surprisingly, good heuristics seem to use these structures early on [16], [17]. A direct consequence of this structural exploitation is a search schedule that spends more time improving the parts of the instance that are likely sub-optimal while keeping intact what is already good [11], [18]. For example, state-of-the-art SAT solvers handle real-world instances with tens of thousands of variables because they are able to recognize the maximally-constrained variables and know when to restart once this recognition is likely to be wrong [12]. We hypothesize that these findings constitute a coherent intellectual basis to study and understand the near-optimal human performance on computationally intractable problems. In particular, the way human problem-solving techniques schedule modifications through sequences of solutions may provide good evidence for their structural exploitation even if the structures are unknown.

In this paper, we provide evidence for this hypothesis by studying problem-solving on the Traveling Salesman Problem (TSP). The use of widely-studied optimization problems to test human problem-solving provides the theoretical and practical background necessary to probe very specific aspects of problem solving. In particular, the TSP seems ideal for our purpose because of the joint interest in optimization and psychology. In its most popular version, it asks to find the shortest tour that passes through a set of points (cities) on the Euclidean plane [5]. In operations research and mathematical programming, it has been one of the most commonly attacked problems because of its many applications in genome sequencing [19], semi-conductor manufacturing, and touring optimization [20]. Consequently, it has been a touchstone of the effectiveness for many popular algorithms (e.g., dynamic programming [21], simulated annealing [22], genetic algorithms [23], neural networks [24])

In psychology, it has drawn interest because of the surprisingly good human performance on it. Additionally, the problem can easily be visualized and understood, and problem-solving seems to involve little cognitive load [25], [26]. Although the good human performance on the TSP has been known for long a time [25], recent studies have shown that this performance is very close to optimal and is competitive with heuristics on relatively small instances [26]–[36]. However, current models of human performance are usually drawn from one trial without feedback. This would be like only analyzing the initial solution of a heuristic search procedure, leaving unclear how well it schedules modifications and hence exploits structure. Although people seem easily to understand the requirements that are necessary to find the optimal solution, such as following the convex hull (the minimum convex set of cities that contain all cities on an instance) and avoiding self-crossing tours [26], [29]–[31], this information is insufficient to determine structural exploitation.

**Figure 1:**
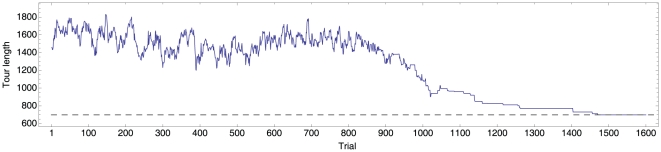
Simulated annealing optimization of instance 22. Solution costs of best run out of 1000 simulations. Solutions traversed are constrained to tours that follow the convex hull and have no self-crossings. (Temperature schedule 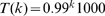).

**Figure 2:**
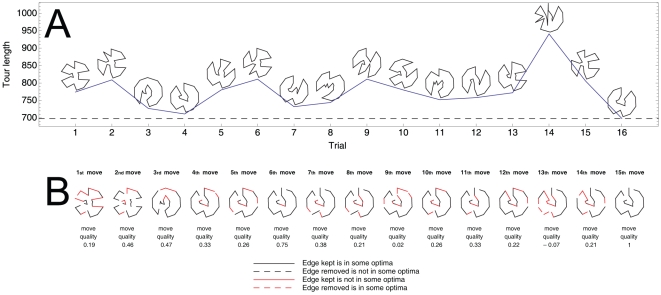
Typical performance of participants on instance 22. A) Participant finds the optimal solution in 16 trials. The solutions and lengths are depicted. B) Move quality for trials 2 and thereafter. For example, the first move shows the modifications performed to the better solution so far (solution of trial 1) to achieve solution of trial 2. As another example, the 6th move shows the modification performed to the better solution so far (solution of trial 4) to achieve solution of trial 7. A good modification (shown in black) is either to keep an edge that belongs to the optimum or to remove an edges not in the optimum. The remaining modifications are shown in red.

Consider, for example, the most basic version of optimization by Simulating Annealing (SA) applied to the TSP, which, while theoretically guaranteed to find the optimal solution provided infinite trials [22], does not exploit structure. A routine run of SA on an instance may take several orders of magnitude longer than humans, even if SA only traverses the space of tours that follow the convex hull and do not have self-crossing. For example, compare the 1600 steps required by a favorable simulation of SA (Fig. 1, see Supporting Information S1 for details) to the much fewer steps typically required by human participants (Fig. 2A for an example) to optimally solve instance 22 of our study. Although participants may make additional mental tours and estimate their costs before actually providing a new solution to the experimenter, it is clear that human problem-solving is taking very efficient shortcuts in solving the TSP, perhaps by exploiting deep structures of the problem. In our simulation, SA does not have any understanding of the structure of the problem beyond following the convex hull and avoiding self-crossings. Good heuristics for the TSP, however, explore the solution space by keeping edges that are likely good while removing the rest [37], which may be a reasonable characteristic of human problem-solving as well.

In this paper, we study data from 28 participants who solved 28 instances of the TSP, were provided feedback and were allowed to solve any instance unlimited times. First, we show that allowing repetitions and feedback significantly helps to improve solutions. Additionally, we show that the human solutions are significantly different from random tours that follow the convex hull and do not have self-crossing. Second, we show that participants schedule modifications so that edges that belong to the optimum are significantly more likely to stay than other edges. Finally, we test for the presence of a significant effect of practice. We show that there is a power-law between total number of trials and participant's performance and that the ability to tell good from bad edges diminishes with more trials.

## Results

**Figure 3:**
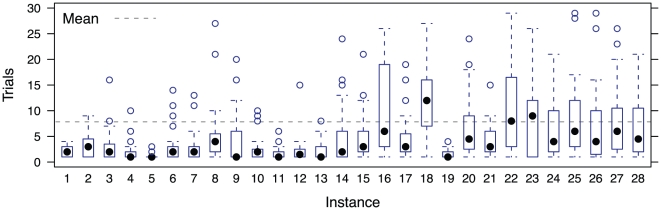
Trials per instance.

We use a confidence level of 95% for all our statistical tests. Twenty-eight participants provided a total of 6441 solutions, with an average of 230.03 solutions per participant (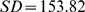, Max 635, Min 39) on 28 instances of the TSP (See Materials and Methods.) The mean practice time was 2.6 hours (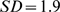) A small percentage (6.7%) of solutions contained self-crossings, which we excluded from analyses [31]. Fig. 3 shows a summary of the number of trials per participant for each instance.

**Figure 4:**
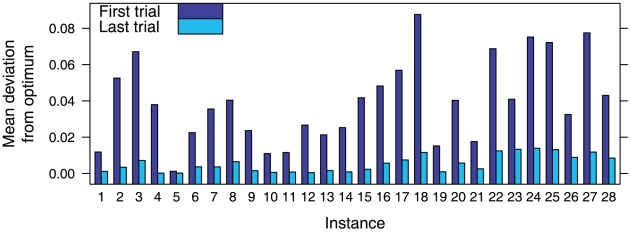
Mean deviation from optimum found by participants in the first and last trials.

We first found that the feedback and repetition allowed participants to improve their solutions significantly. Fig. 4 shows the mean deviation from optima (all participants) for the first and last trials. We used a Welch-Satterthwaite two-sample *t*-test to assess whether the deviation from the optima of the first trial (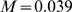, 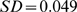, 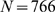) was significantly higher than that of the last trial (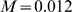, 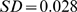, 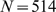) (Notice that the last solution may not be the best solution and that not all participants provided more than 1 trial to all instances.) The improvement was significant, 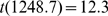, 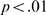.

### Directed search

**Figure 5:**
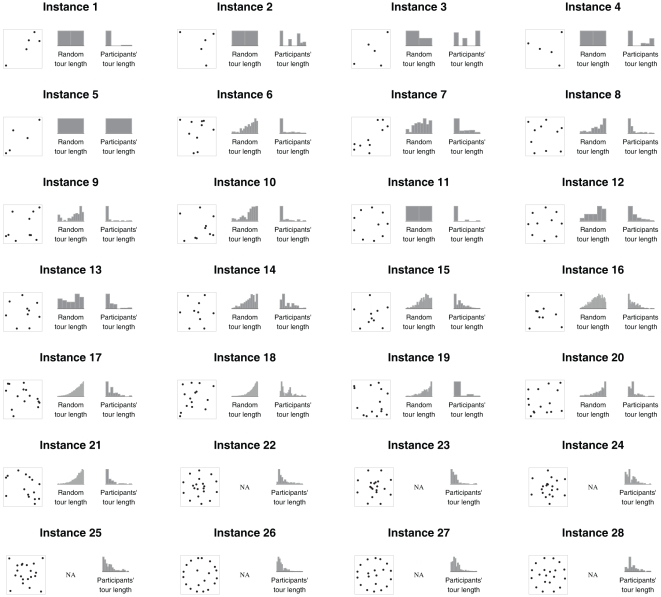
Instances, random length distributions of tours that follow the convex hull and have no self-crossing, and participants' distributions of tour lengths. The random and participants' distributions are significantly different.

We tested whether the solutions provided by participants can be explained as random samples from the distribution of tours that follow the convex hull and do not have self-crossings [30], [31], [35]. For each of the first 21 instances, we compute the distribution of these solutions by enumerating all tours with no self-crossing that have 30% or less deviation from optimum (see Fig. 5). (We did not find feasible to do this test for instances 22 through 28 due to the size of their solution spaces. Even though considering only the tours that follow the convex hull and have no self-crossing dramatically reduces the search space, the number of solutions is still factorial of the number of cities.) For each instance, we pooled solutions provided by participants and computed a 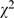 goodness-of-fit test to check whether the participants' distributions of tour lengths were different from those of random solutions. (Notice that this is a more stringent test than checking whether the edges of the tours were similar because several tours may have the same length; our approach decreased the likelihood of rejecting the null hypothesis.) We found this difference to be significant for all instances, 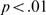, but instance 3, 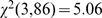, 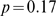.

### Move Quality: Efficient Exploration of Solution Space

We considered a good search procedure to be one that does not waste time trying to optimize parts that are already optimal, producing an efficient exploration of the solution space. We called a *move* a modification to the current better solution during a sequence of trials. We measured the *move quality* by the difference between the proportions of edges kept and removed that belong to the optimal solution. The move quality then is a continuous number between −1 and 1. A move quality from −1 to 0 is considered bad (i.e., good edges are more likely to be removed than bad edges), 0 is random (random modification), and 0 to 1 is good (i.e., good edges more likely to stay than bad edges.) (See Materials and Methods for details.)

**Figure 6:**
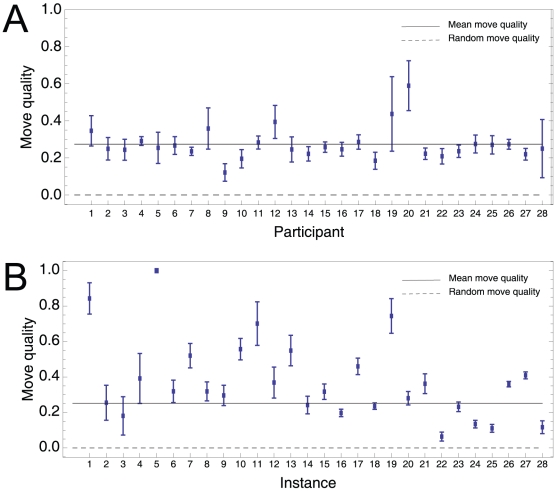
Move quality of participants. The move quality between −1 and 0 indicates a redundant search procedure that wrongly removes edges that are in the optimum (good edges) and keeps edges not in the optimum (bad edges); move quality of 0 indicates random move; move quality between 0 and 1 indicates a move that is more likely to keep good edges than bad edges. The mean move quality across participants and instances is significantly higher than the random move quality (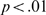) Move quality is more homogenous between participants (A) than participants (B).

Across participants and instances, we found that the move quality, 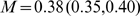, was significantly higher than move quality of a random move (movie quality = 0), 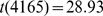, 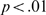. Participants seemed to make purposeful changes to parts of the solution that led to better solutions. By performing a two-way analysis of variance for the effect of instance and participant on move quality, we found that instance, 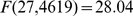, 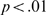, and participant, 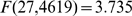, 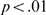, had significant main effects, but there was a larger between-instance than between-participant variability, suggesting that participants had similar search procedures but the structure of some instances might have been harder to exploit than others. Fig. 6A shows the mean move quality per participant; Fig. 6B shows the mean move quality per instance.

### Effect of Trials on Move Quality

We analyzed the effect of trials (within instance) on move quality to understand how the solution space exploration changes with more solution attempts. We assessed the fixed effect of trial on move quality by performing a hierarchical logistic regression, controlling for the random effect of participant and instance on the slope and intercept of the regression. We fitted an overdispersed binomial distribution with a logit link [38] (see Supporting Information S1 for details.)

**Figure 7:**
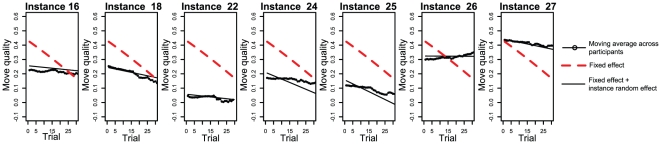
Effect of trials on move quality. The random effect and moving average of the raw data are plotted up to 2 standard deviations (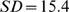) of the number of trials per instance.

In the regression, we expressed the trial predictor in units of 8 trials so that it approximately matched the average number of trials per instance (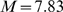, 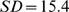). (This will be useful when later we analyze the additional effect of instance difficulty on move quality.) We found a significant negative fixed effect of trial, 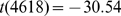, 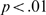, on move quality. There was a maximum of 3.8% (2.6, 4.6) reduction in move quality per each eight trials around the center of the predictor (the center of predictor in eight-trial units is 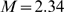, 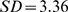) Fig. 7 shows the fixed and random effects of the regression and a moving average of the raw data across participants on the seven instances with larger number of trials.

We performed a second regression to analyze the effect of instance difficulty on move quality. Given that the instances were presented in order of difficulty (see Methods and Materials for details), we used the instance presentation order (i.e., from 0 to 27) as a proxy for its difficulty and assumed that difficulty increased linearly. We developed a hierarchical logistic regression model to assess the fixed main effects of trial and instance difficulty on move quality. We controlled for the random effect of participant on the intercept and slope of trial, and the effect on participant on the slope of instance difficulty. Additionally, we controlled for the random effect of instance on the intercept and slope of trial. This regression allowed to measure the main effects of number of trials and instance difficulty while allowing changes between participants and between instances. (An additional regression ruled out the interaction between number of trials and instance difficulty, 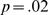)

We found a negative effect of instance difficulty of 0.45% (.03, .05) and a negative effect of eight trials of 2.6% (1.7, 3.5)—around the center of the predictors (instance difficulty: 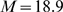; trials 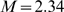). This time, the effect of trials was lower than the previous regression. Given that the measured effect of eight trials can be approximately compared to the effect of solving a harder instance, it could be concluded that the effect of trials was 5.7 times larger (odds ratio: 2.6/0.45) than increasing the instance difficulty. This suggested that the number of trials had a major infuence on the quality of the moves, whereas the difficulty did not.

### Power law of practice vs. performance

**Figure 8:**
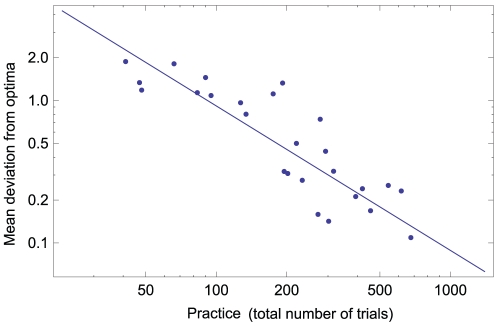
Relationship between practice and performance per participant. There is power law relationship between performance (mean deviation from optima of best solutions) as a function of practice (total number of trials.) (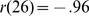, 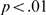).

A Pearson product-moment coefficient was computed to assess the power-law relationship between practice (the total number of trials) and the mean deviation from optima (performance) obtained by each participant. There was a negative correlation between these two variables, 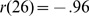, 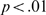 (see Fig. 8.) This is consistent with the effect of trials on move quality. Given that participants were prone to random (i.e., non-directed) modifications at later trials, it was harder for them to reach a better solution and to improve the overall performance measure.

We additionally analyzed whether prior practice had an effect on the cost of the first solution to a previously-unseen instance. We performed a hierarchical logistic regression model considering instances as random intercept and prior practice as a fixed effect on the cost of the first solution to a previously-unseen instance (only instances were used as random effects to improve the precision of the effect measurement [a two-way ANOVA showed a higher variance by instance, 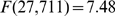, 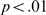, than participant, 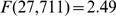, 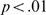]. See Supporting Information S1 for details.) Surprinsingly, the effect of prior practice on the cost of the first solution to a previously-unseen instance was significantly negative, 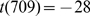, 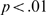; however, this was likely confounded with the effect of the instance difficulty because harder instances were more likely to be preceded by longer prior practice—the easier instances were presented first (See Materials and Methods for details on the Procedure.)

## Discussion

Our results provide evidence that humans (implicitly) know a great deal more about the structure of the TSP than previously shown in the literature. In particular, participants improved their solutions significantly after getting feedback and repetition. A reasonable concern would be that the unlimited repetition would allow participants to search the space of solutions exhaustively. However, we found that participants followed a very directed search pattern. Their solutions were significantly different from random samples of tours that follow the convex hull and do not have self-crossings, a common feature of human solutions [30], [31]. When we analyzed the sequences of solutions, participants focused their search primarily on the sub-optimal parts of the current better solution leaving intact what was already good. This suggests that participants knew distinctive structural properties of the TSP to accurately infer the edges that belong to the optimal solution, paralleling the behavior of heuristics that exploit structures. We found, additionally, that this capacity decayed with more trials, suggesting that participants ran out of ideas and became more exhaustive (i.e., non-directed) toward the end of the search. This is consistent with the power law between practice and performance: it required an increasing number of trials to find a better solution and, therefore, improve performance.

Although our conclusions are based on participants of a long experiment with a large number of opportunities for practicing, we believe our results generalize to the casual subject as well. In a number of previous experiments from the literature, it has been shown that people provide very good solutions to the TSP, even without feedback [26]–[36]. It is plausible that the solutions of these experiments are only a fraction of the solutions that people think are good. In our experiment, the repetition facilitated trying several solutions while feedback indicated which solutions were more desirable.

The quality of the search procedure found in our study makes even more puzzling the question as to why humans are so good at solving the TSP. Previous studies have suggested that the characteristics of the visual system, such as visual acuity and attention, allow to decompose the problem hierarchically and merge subsolutions efficiently [28], [33], [36] or that humans have a natural capacity to assess optimality visually [34]. In our study, it is difficult to reach a more specific answer as to how humans explore the solution space efficiently when feedback and repetitions are allowed, but we believe that people may know structural properties of intractable problems well. We could not conclude that this was a learned capacity through practice wihtin our study; we even found a negative effect of practice on the cost of solutions, which may well be confounded with the effect of instance difficulty. Moreover, we found a very small effect of difficulty on the move quality; this suggests that the capacity to detect good from bad edges is nearly independent of the instances considered in our experiment.

In general, the use of widely-studied optimization problems provides a useful starting point to analyze structure exploitation and how this is learned. Intrinsically-structured problems, such as the popular game Sudoku, are particularly appealing. A generalization of this game, called *quasi-group completion* [39], has already provided a means to studying heuristics that exploit structural properties, and may help to serve the same purpose in psychology.Theoretically, it can be computationally harder to detect structures than to solve the instance itself [40], [41]. However, there is a point where learning these structures is ultimately beneficial in the long-term because most naturally-occurring instances of hard problems are highly structured. It is likely that this kind of structural learning plays a key role in human problem-solving [42], [43].

A general issue is to understand the source of the structure of the typical instance. Important steps have been taken in understanding the “shape” of the solution space of general optimization problems [44] and how easily structures suddenly appear in any given system [45]. This supports the idea that structural discovery is an essential part of human problem-solving.

Finally, we believe our hypothesis and results may release some of the tension between cognitive modelers that consider worst-case intractability a secondary issue (e.g., rational analysis) and those who do not. For example, bounded-rationality theory [46], and the more sophisticated fixed-parameter tractable cognition theory [47] try to put some computational complexity bounds on the computational-level models of behavior. Taking this issue on the grounds of how models can be integrated under a coherent framework that is both flexible and plausible [48], we believe that rational analyses that arrive at wildly worst-case intractable models should not be a big concern because worst cases are uncommon.

## Materials and Methods

### Ethics statement

The present experiment was not submitted for approval to a centralized ethics review board because a committee from the *Departamento de Ingeniería Informática* reviewed the ethical aspects as part of the proposal and defense of one of the author's thesis. Additionally, it was felt that the study involved no more than the reasonable minimal risks that exist in daily life; anticipated benefits for the subjects and the importance of the knowledge expected to be acquired outweighed these risks.

Participants were asked to agree to the terms of an electronic consent form before they could participate in the study. It was explained that their electronic agreement was considered voluntary willingness to take part in the experiment, from which they could drop out at any time without penalty.

### Participants

In this paper, we analyzed twenty-eight participants (2 women, 26 men, mean age = 21.7, SD = 2) who were eligible to go to the finals of a “Traveling Salesman Championship,” in which sixty-eight undergraduate students (4 women, 64 men, mean age = 21.9 years, *SD* = 2.1) from the *Departamento de Ingeniería Informática* of the *Universidad de Santiago*, Chile, volunteered to participate by responding to flyers posted on the Department's news board and a web banner in one of the authors' home page. To be eligible to go to the Finals, a participant had to provide solutions of at most 5% deviation from optimum for each of 28 instances of the Traveling Salesman Problem (TSP); we analyze the solutions provided for these instances. There were prizes awarded to the three best participants of the championship, who provided the best overall solutions to all instances of the finals. Participants were treated in accordance with the “Ethical Principles of Psychologists and Code of Conduct” [49] and local regulations of the Universidad de Santiago and the Ministry of Education of Chile.

### Materials

#### Game

The experiment was presented as a game-like Adobe Flash application [50] embedded on a web page. Once the player logged on to the system, the game forced full-screen game playing and kept the playable area at 800×600 pixels.

**Figure 9:**
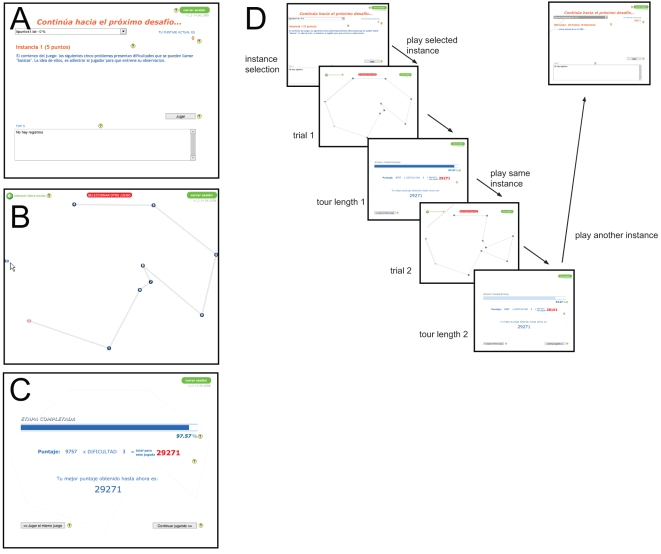
Game to capture human problem-solving on the Traveling Salesman Problem. A) Instance selection screen. B) Tour construction screen. C) Results screen. D) A typical game session. An instance is solved several times until the optimum is found, a 5% or less of deviation from optimum is found, or the player decides to play a previously solved instance.

The application presented the “lobby”, “game play,” and “results” screens. The first screen, the “lobby” (Fig. 9A), showed the participant's position in the general rankings, a pop-down menu with the list of instances available to solve, and a centered text area about the instance currently selected on the pop-down menu that described the number of cities, relative difficulty, and some historical background. To start playing, the participant had to click on “Play” button. There was another button to close the application.

The second screen (the “gameplay”, Fig. 9B) showed the actual instance to solve. The cities were shown as rotating blue ellipses. The participant would make a tour by sequentially clicking cities on the screen. An edge was shown as a thick light gray line connecting the cities. The cities that were currently part of the tour would stop rotating and turn gray. The last city clicked, and from which the tour would continue, was shown in red. Once the last city of the instance was selected, the application would automatically complete the tour (i.e., the participant did not need to select the first city again.) At any time, the participant could press an “undo” button on the top-left corner of the screen that recursively removed the last city clicked. It was not possible to exit the application at the gameplay screen unless the web browser was manually shut down. The application remotely recorded the time spent solving an instance, the sequence of points selected, and the undo actions.

During the gameplay screen, the participant's account was locked to prevent practice without recording in other computers. After recording a complete solution, the account would be unlocked. If the game were forced to close during “gameplay,” the participant would be unable to log in again, forcing him or her to contact the researcher to assess the situation.

Once the instance was solved, the third screen (the “results”, Fig. 9C) showed the solution's deviation from the optimum as a percentage. Messages with sounds would appear if the solution found was the best yet found by the individual participant or between participants. If the solution found were the optimum, a message would congratulate the participant. If the solution found had a deviation larger than 5%, the participant would not be allowed to advance to the next instance. Unless the optimum was found, a button would allow the participant to play the same instance immediately. Another button would take the participant to the “lobby.” A typical game session is shown in Fig. 9D.

#### Instances

Instances 1 through 10 and 17 through 21 (see Fig. 5) were extracted and scaled from [32]. The other 13 instances (Fig. 5) were extracted from [26]. For instances 1 through 21, we computed all solutions with up to 30% deviation. For the rest of the instances, we computed the optimal solution with the Concorde solver [20].

The game presented the instances in order of increasing difficulty. The difficulty was assessed based on the time it took the authors and the Concorde solver to solve them optimally [20].

### Procedure

We allowed a registration period of two weeks prior to the beginning of the championship. Participants would register and read an online consent form. We asked them to provide an alias to be used online and an email for follow-up. We published the list of players online before the championship started. The experiment lasted 14 days (from one Sunday midnight to another Sunday midnight.) The ranking was manually updated every two days because we wanted to balance the need for solitary practice and competition against others.

### Measures of practice, performance, and move quality

#### Practice and performance

Practice was measured as the total number of trials across instances. The cost of a solution for an instance was measured as the deviation from the instance's optimum. The performance of a participant on an instance was measured as the cost of his or her best solution for that particular instance. The general performance of a participant was measured as the participant's mean performance on all the instances. The performances of participants were used to rank them and give prizes.

#### Move Quality

An instance of a TSP problem is defined as (1) a set of tours 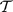 and (2) a tour length function 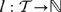 which is computed as the sum of Euclidean distances, rounded to the nearest integer, between the cities of the tour [51]. A solution 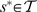 with 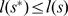 for all 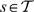 is called a global optimum or simply optimum.

A search procedure traverses the solution space 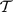 through a series of solutions
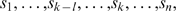
from the initial solution 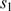 to the final solution 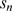. Let 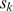 be an intermediate solution. Without lost of generality, let 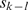 be the best solution found prior to 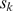 (i.e., 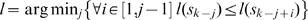). We consider 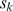 an attempt to improve 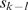. A *move* 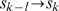 contains the modifications performed to 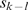 to reach 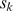. Notice that a move only depends on the intermediate solution 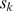 and the sequence of solutions 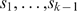 from which the solution 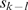 can be determined.

We propose the *move quality* as a measure that assesses the degree to which the modifications made to the previously better solution are aimed at correcting sub-optimal edges while retaining what is already good. A simple definition of what is good are the edges that appear in all optimal solutions, and consequently, bad edges are those edges that do not appear in any optimal solution. The edges that appear in all optimal solutions are called the *backbone* [37], [40]. Incidentally, the relative size of an instance's backbone is a good measure of its difficulty [52], [53].

Let 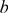 be the backbone of an instance, 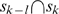 be the set of edges that are kept between solutions 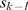 and 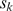, and 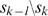 the set of edges that are removed from 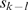 in solution 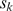. We want the edges kept to be more likely to be part of the backbone than the edges removed. Because few edges are removed at each move, one way of capturing this intuition is by comparing the *proportion* of correctly kept and removed edges. Let 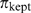 be 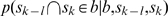 and 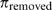 be 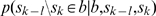, the proportion of edges kept and removed that belong to the backbone, respectively. We define the *move quality* as the difference 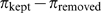 of these two proportions. This measure varies from −1 to 0 (bad move; the proportion of good edges removed is larger than the proportion of good edges kept), 0 (random move), and 0 to 1 (a good move; the proportion of good edges kept is larger than the proportion of good edges removed.) The confidence interval for 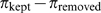 can be easily obtained [54] by
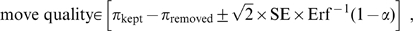
where
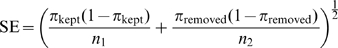
is the standard error, 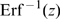 is the inverse of the Gaussian distribution integral
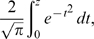


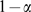
 is the confidence level, 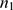 and 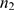 are the number of edges kept and removed, respectively.

## Supporting Information

Supporting Information S1 Simulations and Regressions.
(0.18 MB PDF)
