# Supplementary material for: People Efficiently Explore the Solution Space of the Computationally Intractable Traveling Salesman Problem to Find Near-Optimal Tours
Source: PLoS One. 2010 Jul 29;5(7):e11685. doi: 10.1371/journal.pone.0011685 (PMC2912227; doi:10.1371/journal.pone.0011685)
Supplement: Supporting Information S1 — Simulations and Regressions. (0.18 MB PDF) [file pone.0011685.s001.pdf]

Supporting Information of "People efficiently explore the solution space of the computationally intractable Traveling Salesman Problem to find near-optimal tours"

Daniel E. Acuña
Department of Computer Science and Engineering
University of Minnesota, Twin Cities
Minneapolis, Minnesota, USA

Víctor Parada
Departamento de Ingeniería Informática
Universidad de Santiago
Santiago, Chile

1 Simulated Annealing Optimization Simulation

Simulated Annealing (SA) is a stochastic general-purpose global optimization procedure inspired by annealing in metallurgy, where a material is heated and then slowly cooled so that the size of its crystals is maximized and its defects minimized [1]. SA requires the definition of a *neighborhood* of solutions around any given solution and a stochastic "jump" to a new solution in this neighborhood if this new solution is better. However, if the new solution is worse, SA can still jump, with a probability that depends on the difference between the cost of the current solution, the new solution, and a *temperature* parameter $T(k)$, where k stands for the trial.

We use the following parameters for our simulation. We define a neighborhood $N(s)$ around the solution s as any tour s' that can be obtained by reversing the traveling order between two randomly-selected cities on the tour s. For example, we can reverse the cities 2 and 4 on the tour $s =< 1,\mathbf{2},\mathbf{3},\mathbf{4},5 >$ and obtain $s' =< 1,\mathbf{4},\mathbf{3},\mathbf{2},5 >$. Additionally, we require all neighbors to follow the convex hull and no self-crossing. If solution s' follows the convex hull, has no self-crossing and can be obtained from some s by reversing a subtour, then $s' \in N(s)$.

The probability of jumping to a new candidate solution s' from the current solution s is 1 if the tour length of the new solution $l(s')$ is less than the tour length of the current solution $l(s)$, or the probability is $\exp((l(s) - l(s'))/T(k))$

otherwise. This ensures that the probability of accepting a worse solution decreases with time, ultimately reaching zero in the limit. We choose the cooling schedule $T(k) = \alpha^k T_0$, where $T_0 = 1000$ and $\alpha = 0.99$. Simulated annealing has been proven to find the optimum with probability 1 if infinite trials are performed. We ran 1000 simulations of our simulated annealing algorithm on Instance 22 for 10,000 trials. Only 1% of simulations found the optimal solution on the first 2000 trials. On Fig. 2 of the main text, we display the best simulation, which found the optimal solution at trial 1480. We performed additional simulations with different T_0 and $\alpha = 0.9, 0.95$, and a neighborhood using the Lin-Kernighan swaps [2], which do not check for self-crossing or convex hull. However, we did not find a simulation with better overall performance than the one we previously describe.

2 Model of the Effect of Practice on Performance

We developed a hierarchical logistic regression model [3] considering instances as random intercepts and practice (total number of trials) as a fixed effect on the quality of the first solution to an instance. We apply a logarithmic transformation to the practice variable to make it more normal-like and then we center it to improve the regression:

$$z_i = \log\left(\text{practice}_i\right) - \text{mean}\left(\log\left(\text{practice}\right)\right). \tag{1}$$

Moreover, we transform the deviation from optimum to lie between 0 and 1 (Eq. 2). Finally, the model we fit is as follows

$$\text{deviation from optimum}_i = p_i^{-1} - 1 \tag{2}$$

$$p_i = \text{logit}^{-1}(\pi_i) \tag{3}$$

$$\pi_i = \alpha_{j[i]} + \beta_i z_i \tag{4}$$

where i is the ith data-level of information about the first solution provided to instance j with mean-centered logged practice z_i. Instance j acts as a random effect on the intercept

$$\alpha_j \sim \text{N}\left(\mu_\alpha, \sigma_\alpha\right), \tag{5}$$

and we estimate the effect of practice

$$\beta_i \sim \text{N}(\mu_\beta, \sigma_\beta). \tag{6}$$

We obtain the following estimations: $\hat{\sigma}_\pi^2 = 0.001$, $\hat{\sigma}_\alpha^2 \approx 0$, $\hat{\mu}_\alpha = 3.12$, $\hat{\mu}_\beta = -.17$, and $\hat{\sigma}_\beta^2 \approx 0$. The effect of practice ($\hat{\mu}_\beta = -.17$) is significantly negative.

3 Model of the Effect of Trials on Move Quality

We perform a hierarchical logistic regression model [3] to measure the effect of trial on move quality. We first transform move quality so that it lays between 0 and 1 (Eq. 7). The resulting regression is

$$\text{move quality}_i = 2 \times p_i - 1 \tag{7}$$

$$p_i = \text{logit}^{-1}(\pi_i) \tag{8}$$

$$\pi_i = \alpha_{j[i],k[i]} + \beta_{j[i],k[i]} z_{8i}, \tag{9}$$

where z_{8i} is the mean-centered predictor in 8-trial units within instance and participant, j is the instance's and k is the participant's random effect, respectively. An explicit view of the random and fixed effects is

$$\begin{pmatrix} \alpha_{j,k} \\ \beta_{j,k} \end{pmatrix} = \begin{pmatrix} \mu_0 \\ \mu_1 \end{pmatrix} + \begin{pmatrix} \gamma_{0j} \\ \gamma_{1j} \end{pmatrix} + \begin{pmatrix} \gamma_{0k} \\ \gamma_{1k} \end{pmatrix}, \tag{10}$$

with

$$\begin{pmatrix} \gamma_{0j} \\ \gamma_{1j} \end{pmatrix} \sim \text{N}\left(\begin{pmatrix} 0 \\ 0 \end{pmatrix}, \begin{pmatrix} \sigma_{0j}^2 & \rho_j \sigma_{0j} \sigma_{1j} \\ \rho_j \sigma_{0j} \sigma_{1j} & \sigma_{1j}^2 \end{pmatrix} \right) \tag{11}$$

$$\begin{pmatrix} \gamma_{0k} \\ \gamma_{1k} \end{pmatrix} \sim \text{N}\left(\begin{pmatrix} 0 \\ 0 \end{pmatrix}, \begin{pmatrix} \sigma_{0k}^2 & \rho_k \sigma_{0k} \sigma_{1k} \\ \rho_k \sigma_{0k} \sigma_{1k} & \sigma_{1k}^2 \end{pmatrix} \right). \tag{12}$$

We found that $\left(\begin{smallmatrix} \mu_0 \\ \mu_1 \end{smallmatrix}\right) = \left(\begin{smallmatrix} .56 \\ -.15 \end{smallmatrix}\right)$, $\hat{\sigma}_{0j}^2 = .002$, $\hat{\sigma}_{1j}^2 = .0004$, $\rho_j = -.48$, $\hat{\sigma}_{0k}^2 = .0002$, $\hat{\sigma}_{1k}^2 \approx 0$, $\rho_k = .59$, and $\hat{\sigma}_{\text{move quality}}^2 = .0256$. The effect of trials on move quality ($\mu_1 = -.15$) is significant, $p < .01$. The model fits well across the prediction (see binned residual plot in Fig. 1)

Now, we describe the second regression that adds the fixed effect of instance difficulty on the move quality while controlling for the random effect of instance and participant on the slope of effect of instance difficulty.

Let the predictor d_j be the mean-centered difficulty of an instance, where the mean of the predictor was around $M = 19.94$. Given that the experiment presented the instance in order of difficulty, we assume a linear increase in difficulty. The regression performed was as follows

$$\pi_i = \alpha_{j[i],k[i]} + \beta^0_{j[i],k[i]} z_{8i} + \beta^1_{k[i]} d_{j[i]}, \tag{13}$$

where $d_{j[i]}$ is the difficulty of the instance being solved in move i. The explicit view of the random and fixed effects is

$$\begin{pmatrix} \alpha_{j,k} \\ \beta^0_{j,k} \\ \beta^1_{k[j]} \end{pmatrix} = \begin{pmatrix} \mu_0 \\ \mu_1 \\ \mu_2 d_j \end{pmatrix} + \begin{pmatrix} \gamma_{0j} \\ \gamma_{1j} \\ 0 \end{pmatrix} + \begin{pmatrix} \gamma_{0k} \\ \gamma_{1k} \\ \gamma_{2k} \end{pmatrix},$$

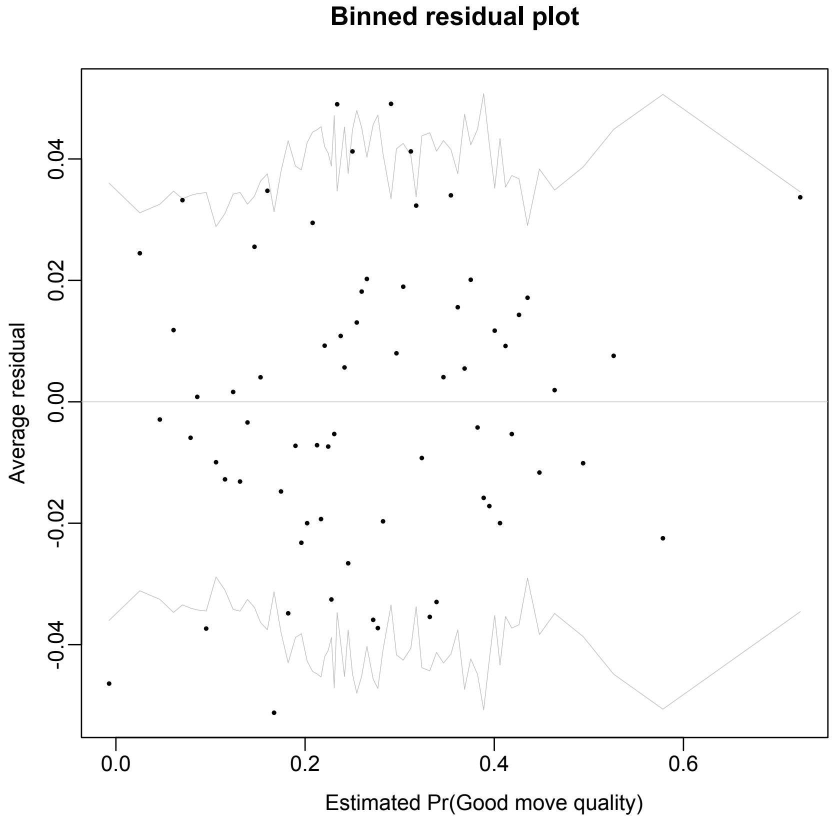

Figure 1: Binned residuals with 95% confidence interval of hierarchical logistic mixed-effect regression for the effect of trial on the move quality

with

$$\begin{pmatrix} \gamma_{0j} \\ \gamma_{1j} \end{pmatrix} \sim \mathrm{N}\left(\begin{pmatrix} 0 \\ 0 \end{pmatrix}, \begin{pmatrix} \sigma_{0j}^2 & \rho_j \sigma_{0j} \sigma_{1j} \\ \rho_j \sigma_{0j} \sigma_{1j} & \sigma_{1j}^2 \end{pmatrix} \right) \tag{14}$$

$$\begin{pmatrix} \gamma_{0k} \\ \gamma_{1k} \\ \gamma_{2k} \end{pmatrix} \sim \mathrm{N}\left(\begin{pmatrix} 0 \\ 0 \\ 0 \end{pmatrix}, \begin{pmatrix} \sigma_{0k}^2 & \rho_{0k} \sigma_{0k} \sigma_{1k} & \rho_{1k} \sigma_{0k} \sigma_{2k} \\ \rho_{0k} \sigma_{0k} \sigma_{1k} & \sigma_{1k}^2 & \rho_{2k} \sigma_{1k} \sigma_{2k} \\ \rho_{1k} \sigma_{0k} \sigma_{2k} & \rho_{2k} \sigma_{1k} \sigma_{2k} & \sigma_{2k}^2 \end{pmatrix} \right). \tag{15}$$

We are interested in the fixed effects μ_0, μ_1 and μ_2. The following are the estimates: $\hat{\mu}_0 = 0.56$, $\hat{\mu}_1 = -.11$, and $\hat{\mu}_2 = -.02$. The errors are $\hat{\sigma}_{0j}^2 = .0024$, $\hat{\sigma}_{1j}^2 = .0002$, $\hat{\rho}_j = -.49$, $\hat{\sigma}_{0k}^2 = .0003$, $\hat{\sigma}_{1k}^2 \approx 0$, $\hat{\sigma}_{2k}^2 \approx 0$, and $\hat{\rho}_{0k} = .74$, $\hat{\rho}_{1k} = -.39$ and $\hat{\rho}_{2k} = -.49$. Finally, $\hat{\sigma}_{\text{move quality}}^2 = 0.0225$

References

[1] S. Kirkpatrick, D. G. Jr., and M. P. Vecchi, "Optimization by simmulated annealing," *Science*, vol. 220, no. 4598, pp. 671–680, 1983.

[2] S. Lin and B. W. Kernighan, "An effective heuristic algorithm for the traveling-salesman problem," *Operations Research*, vol. 21, no. 2, pp. 498–516, 1973.

[3] A. Gelman and J. Hill, *Data analysis using regression and multilevel*, ch. Multilevel logistic regression, pp. 301–323. Cambridge ; New York: Cambridge University Press, 2007.
